# RTMS parameters in tinnitus trials: a systematic review

**DOI:** 10.1038/s41598-019-48750-9

**Published:** 2019-08-21

**Authors:** Stefan Schoisswohl, Kushal Agrawal, Jorge Simoes, Patrick Neff, Winfried Schlee, Berthold Langguth, Martin Schecklmann

**Affiliations:** 1 Department of Psychiatry and Psychotherapy, University of Regensburg, Regensburg, Germany; 2 Institute of Databases and Information Systems, University of Ulm, Ulm, Germany; 3 University Research Priority Program “Dynamics of Healthy Aging”, University of Zurich, Zurich, Switzerland; 4 European School for Interdisciplinary Tinnitus Research (ESIT), Regensburg, Germany

**Keywords:** Neuroscience, Neuroscience, Neurology, Neurology

## Abstract

Over the past few years extensive body of research was produced investigating the effects of repetitive transcranial magnetic stimulation (rTMS) for the treatment of chronic tinnitus with heterogeneous results. This heterogeneity is exemplified by two recently published large-scale clinical trials reporting different outcomes. Technical aspects of rTMS were suspected as a potential source for this incongruency. The aim of this systematic review is to examine the overall efficacy as well as to identify possible technical factors relevant for the effectiveness of rTMS tinnitus trials. Via a literature search appropriate original research papers were identified and rTMS parameters were extracted from each study arm for subsequent statistical analysis with respect to observed effects (significant vs. not significant pre-post rTMS effects). Our findings indicate that verum rTMS is superior to sham rTMS as demonstrated by the proportion of significant pre-post contrasts. Some relevant rTMS parameters (e.g., pulse waveform) are not reported. Lower rTMS stimulation intensity was associated with significant effects in verum rTMS arms. An additional stimulation of the DLPFC to the temporal cortex was not found to promote efficacy. Future research should consider differential effects of rTMS induced by technical parameters and strive for an exhaustive reporting of relevant rTMS parameters.

## Introduction

Chronic subjective tinnitus is defined as the perception of a sound, such as ringing or buzzing, without the presence of an external or internal source^1^ with a duration of at least three months^2^. Approximately 10–15% of people living in industrial countries are affected by such persistent sounds and up to now, there is no available cure^3^. Etiology of tinnitus seems to be very heterogeneous, though in most cases it occurs after cochlear damages following noise trauma or hearing loss in general^4^. It is assumed, that as a consequence of diminished or missing acoustic input and the ensuing deprivation of neural input in the auditory pathways, pathological brain changes occur and the “phantom sound”, called tinnitus is generated^5,6^. From a neurophysiological perspective subjective tinnitus is therefore associated with altered neural activity along the auditory pathway^7^ and hyperactivity in auditory brain areas^8,9^ as well as non-auditory brain areas^10^. As noted by Theodoroff and Folmer^11^, these given pathological neural circumstances represent a significant leverage point for the application of recent neuromodulation techniques, in particular repetitive transcranial magnetic stimulation (rTMS).

15 years ago, low-frequency rTMS of the left auditory cortex was introduced as a new possibility to treat tinnitus based on the rationale to reduce the over-activated left temporal cortex^12,13^. Since that time a bulk of trials and also several reviews were published with heterogeneous evaluation of the putative efficacy of rTMS for the treatment of tinnitus. The findings and conclusions of clinical trials with rTMS in tinnitus manifest to be diverse and are particularly denoted with e.g. high interindividual variability, a lack of sham-controlled trials and small effect sizes^14–16^. An early review from Langguth *et al*. in 2008 resumed a “promising potential of rTMS for therapeutic management of tinnitus”^17^. This conclusion is supported by other reviews, which report rTMS as a new therapeutic tool for tinnitus^15^, with potential efficacy^18^, some given evidence^19^ or even significant medium to large effect sizes as shown by a meta-analysis^20^. Furthermore, left temporal low frequency rTMS was declared with a Level C recommendation (possible efficacy) in a consensus statement^21^. Other reviews indicated “very limited support for the use of low-frequency rTMS for the treatment of patients with chronic tinnitus”^22^ or a general tendency to not recommend rTMS for tinnitus^23^.

Beside heterogeneity in the evaluation of the efficacy of rTMS one aspect past reviews have in common, is a demand for the implementation of randomized, sham-controlled clinical trials with an appropriate sample size. To the best of our knowledge, two such trials were conducted and published. The ongoing discourse regarding rTMS in tinnitus proceeds by these two clinical trials with an almost identical methodological design and different reported results. Folmer and colleagues^24^ were able to show a significant effect of a sham-controlled 1 Hz rTMS protocol over the auditory cortex on the improvement of tinnitus severity in a study with 64 patients. In contrast, a recent published multi-center study from Landgrebe *et al*.^25^ involving 146 patients could not report any improvements as a consequence of rTMS even by investigating a larger sample. It was discussed in subsequent letters to the editors, that differences in samples (e.g., sample size), used trial design (e.g., outcome measures), but also technical parameters of rTMS (e.g., TMS devices) might be responsible for the conflicting results^26,27^. In the case of used TMS devices the direction of current flow differs by default^28^, which was shown to be critical for the induction of neuroplasticity^21,29^. Parameter space of technical aspects of rTMS is very large and all of them seem to be relevant for neurophysiological effects of rTMS^30^. As early findings indicate and thus as a rule of thumb, low stimulation frequency decreases (≤1 Hz) and high stimulation frequency increases cortical excitability (≥5 Hz)^31,32^. In the same manner, the number of pulses delivered per session or the stimulation intensity used, might be essential for the effectiveness of rTMS^33,34^.

Even if there are plenty of previous reviews, they remain narrow in focus dealing only with effectiveness in general or just patient characteristics, but do not take the rTMS parameters into account. Hence, the aim of this systematic review is to examine previous research concerning daily rTMS in tinnitus to present a statistical overview of general effectiveness of rTMS as indicated by verum-sham contrast and to investigate the influence of rTMS parameters on the effect of verum rTMS in tinnitus.

## Materials and Methods

### Protocol and registration

The review for this paper was conducted according to the guidelines for “Preferred Reporting Items for Systematic Reviews and Meta-Analysis” (PRISMA^35^; see Supplemental Material 1). Moreover, the details of the protocol for this review were registered in the International Prospective Register of Systematic Reviews, PROSPERO (CRD42018099744).

### Search strategy, study selection and data collection

**Figure 1:**
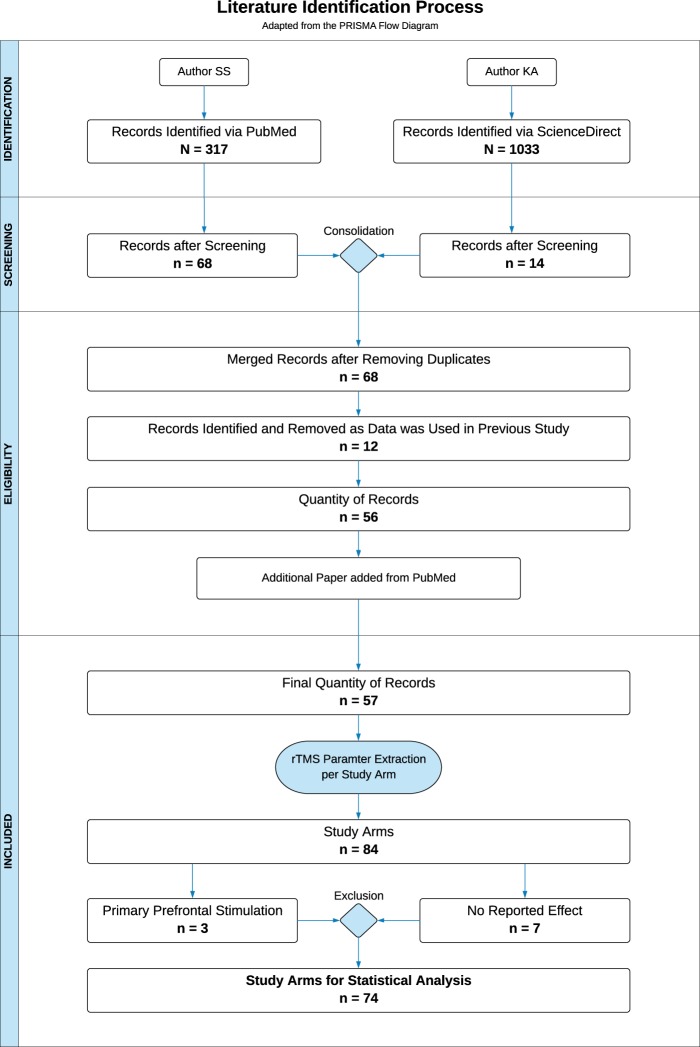
Review Procedure. The procedure of this systematic review from literature identification to the final number of verum rTMS study arms for statistical analysis is depicted as a flow diagram adapted from the PRISMA guidelines^35^.

A systematic literature search was conducted in May 2018 by two independent individuals using the electronic research databases “PubMed” and “ScienceDirect” with the keywords “tinnitus” and “transcranial”. Figure 1 provides an overview of the literature identification process by the means of an adapted flow diagram originally provided by the PRISMA guidelines^35^. In order to identify a maximum quantity of research papers, the keywords were applied to all possible search fields. The utilization of “PubMed” resulted in N = 317 potential papers, the search with the database “ScienceDirect” was able to find N = 1.033 articles. An initial screening of the search outcomes was executed by examining the title as well as the corresponding abstracts with respect to previously defined inclusion criteria: original research data; application of rTMS; repeated sessions; focus on chronic tinnitus. N = 68 from PubMed and n = 14 from ScienceDirect appropriate research papers could be identified. Hereinafter, the results of both search engines were checked for duplicates and merged to a consolidated table with n = 68 articles with all publications of the ScienceDirect search included in the PubMed search. In a next step, the papers were resurveyed in full text, to ascertain, if the studies used data which was already published. As a result, n = 12 papers had to be excluded from our review. Towards the end of the literature identification process, an additional paper was added as detected by regular PubMed searches. Consequently, the final quantity of records for this review and subsequent analysis consisted of n = 57 research papers. In order to deploy statistical analysis, important rTMS parameters were extracted from the records for each verum study arm separately. Thus, the parameter extraction of one single paper could eventuate in multiple study arms for our analysis. Sham arms were not included as we were interested in the effects of rTMS parameters on treatment efficacy. Number of verum and sham arms were counted for estimation of overall rTMS efficacy (see statistics).

Parameters of interest were: manufacturer of the TMS device; type of TMS device; pulse waveform; coil type; coil orientation; stimulation position; stimulation laterality of auditory cortex stimulation (unilateral or bilateral); unilateral stimulation index (extracted by a calculated ratio for the stimulated hemisphere (left = 0, right = 1)); the definition of the stimulated hemisphere with respect to tinnitus laterality; stimulation frequency (Hz) (condensed to inhibitory or excitatory frequency protocols for analysis); stimulation intensity (% of motor threshold); mean motor threshold (%); motor threshold determination method (electromyography (EMG)/visual); number of sessions; overall pulses per session over auditory and prefrontal cortical areas; pulses per session over auditory cortical areas; overall total pulses over auditory and prefrontal cortical areas (calculated as the number of pulses per session * number of sessions); total pulses over auditory cortical (defined as the number of auditory pulses per session * number of sessions); use of a neuronavigation system (yes/no); additional stimulation (e.g., frontal stimulation additionally to auditory cortex stimulation; stimulation parameters were extracted equally; cf. Supplemental Material 2). Further study-specific information like type of additional treatment, study design or used outcome measurements were also extracted from the records, simply to provide an overview and were not considered for statistical analysis.

As no study arm reported the used pulse waveform, we decided to perform statistical analysis for this purpose with the default waveform of the used TMS devices. The information was gathered from user manuals and by contacting the manufacturers of the devices or the authors of the papers, respectively. Our dependent variable of interest was the reported effect of each of the study arms dichotomized to “significant” and “not significant”, as most papers did not report effect sizes. Since only N = 38 study arms provided information about the definition of a primary outcome instrument, a study arm was declared as significant, if 50% or more of the used outcome measurements were reported as “significant”. In case of a lack of information concerning relevant parameters or outcomes, the term “not reported” was used and was not considered in the definition of the reported effect.

### Data analysis

Statistical analysis was performed with the statistic software R (R version 3.4.3; R Foundation for Statistical Computing, Austria; packages “tidyverse and “gmodels”) and focused on the change of symptoms from pre to post treatment of each extracted study arm. For all analyses, we concentrated on trials using stimulation of the temporal or temporoparietal cortex as most studies primarily stimulated these areas (for details see results section) and thus stimulation positions restricted solely to areas outside the temporal region were excluded (three study arms including prefrontal cortex). For seven study arms no information was provided about whether pre to post rTMS changes were significant or not. Therefore, n = 10 study arms were excluded, resulting in 74 study arms included in statistical analyses (cf. Fig. 1). Missing values, “not-reported” effects or information only provided in certain ranges (e.g., pulses per session 1800–3000) were excluded for each parameter analysis individually. Parameters were not analyzed, if 30% of the investigated arms did not provide data. Associations of categorical data with the reported effects (significant vs. not significant) were calculated with χ^2^-tests and Fisher’s exact tests in the case of cell frequencies below 5. To evaluate differences in parametric variables regarding the given effect, Mann-Whitney U-tests for independent samples were computed. Significance level was defined as p ≤ 0.05 and reported uncorrected for multiple comparisons.

## Results

**Figure 2:**
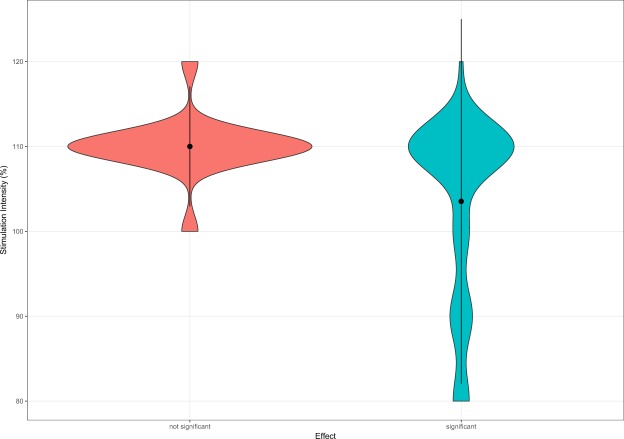
Reported Effect & Stimulation Intensity. The distribution of the stimulation intensity used by study arms separated for the reported results (significant effects N = 51; not significant effects N = 17).

The extraction of relevant parameters from N = 57 research papers resulted in overall N = 74 study arms for which statistical comparisons from pre to post rTMS treatment were done. A detailed overview of the descriptive statistics of rTMS parameters of the single study arms can be found in Supplemental Material 2. Out of 74 study arms used for statistics, 56 arms reported significant effects (76%). In order to ascertain whether the efficacy of verum rTMS is higher in contrast to sham rTMS, we statistically compared the quantity of reported significant and not significant results of verum study arms with those of available 22 sham arms (5 significant; 23%). A χ^2^-test indicated a significant association of the type of study arm (verum or sham) and rTMS efficacy (pre-post change significant or not) showing a superiority of verum in contrast to sham rTMS (p < 0.05). Table 1 shows the results of the association of technical rTMS parameters with efficacy as indicated by pre-post changes in verum study arms. Analyses of parametric data revealed that the group of significant study arms, showed lower stimulation intensity (about 6.5% stimulator output) in contrast to not significant arms (cf. Fig. 2). Out of 18 study arms utilizing a stimulation intensity lower than 110%, 94.44% reported significant results. Whereas, in case of ≥110% stimulation intensity (two study arms with 120% stimulation intensity; one significant, one not significant), 68.00% of 50 study arms state significant findings.” To exclude a potential confounder caused by studies applying continuous theta burst stimulation (cTBS) using commonly lower stimulation intensities, we investigated a possible association of cTBS or rTMS with reported effects. Our results show no significant association (proportion of significant studies did not differ between cTBS and rTMS studies; cf. Table 1), indicating an exclusion of this potential bias in our stimulation intensity results.

**Table 1:** Descriptive and statistical data of 74 study arms.

rTMS Parameter	Significant Study Arms	Not significant Study Arms	p
	n = 56 (75.68%)	n = 18 (24.32%)	
	N (%)	N (%)	
Manufacturer TMS device			
Magstim	14 (77.78)	4 (22.22)	
MagVenture/Medtronic	34 (72.37)	13 (27.66)	0.76
Default waveform			
Biphasic cosine	16 (80.00)	4 (20.00)	
Biphasic sine	36 (72.00)	14 (28.00)	0.56
Stimulation position			
Temporal cortex	32 (78.05)	9 (21.95)	
Temporo-parietal cortex	23 (71.88)	9 (28.12)	0.54
Auditory cortex stimulation laterality			
Bilateral	5 (66.67)	1 (16.67)	
Unilateral	50 (74.73)	17 (25.37)	>0.99
Auditory cortex stimulation hemisphere			
Left	36 (70.59)	15 (29.41)	
Other (contralateral, ipsilateral, bilateral, left or right)	19 (86.36)	3 (13.64)	0.24
Stimulation frequency			
Inhibitory (1 Hz, cTBS)	49 (73.13)	18 (26.87)	
Excitatory (10 Hz, 25 Hz)	6 (100.00)	0 (0.00)	0.33
Stimulation type			
cTBS	4 (80.00)	1 (20.00)	
rTMS (1 Hz, 10 Hz, 25 Hz)	50 (73.53)	18 (26.47)	0.33
Motor threshold determination method			
EMG	36 (75.00)	12 (25.00)	
Visual	10 (76.92)	3 (23.08)	>0.99
Neuronavigation			
Yes	17 (73.91)	6 (26.09)	
No	38 (76.00)	12 (24.00)	0.85
Prefrontal stimulation in addition to auditory cortex stimulation			
Additional prefrontal stimulation	9 (56.25)	7 (43.75)	
No additional prefrontal stimulation	46 (80.70)	11 (19.30)	0.04*
	**M ± SD**	**M ± SD**	**p**
Stimulation hemisphere - auditory cortical areas (left = 0; right = 1)	0.12 ± 0.22 (n = 49)	0.07 ± 0.21 (n = 17)	0.66
Stimulation intensity (%)	103.53 ± 10.7 (n = 51)	110.00 ± 3.54 (n = 17)	0.02*
Number of sessions	8.47 ± 3.38 (n = 55)	9.22 ± 4.76 (n = 18)	0.80
Overall pulses per session − auditory and prefrontal cortical areas	1807.64 ± 838.04 (n = 55)	2488.89 ± 1095.92 (n = 18)	0.03*
Pulses per session − auditory cortical areas	1624.00 ± 671.85 (n = 55)	1877.78 ± 662.04 (n = 18)	0.21
Overall total pulses − auditory and prefrontal cortical areas (pulses per session × number of sessions)	16003.64 ± 11016.50 (n = 55)	22333.33 ± 13266.50 (n = 18)	0.07
Total pulses − auditory cortical areas (pulses per session × number of sessions)	14485.45 ± 9962.53 (n = 55)	17277.78 ± 9730.52 (n = 18)	0.20

*p ≤ 0.05.

With respect to number of pulses, significant study arms used less pulses. Since the number of pulses was only significant for overall pulses per session, we compared the reported effects of study arms which used an additional DLPFC stimulation (n = 16) with those free of any additional stimulation, as DLPFC stimulation in addition to auditory cortex stimulation might result more applied pulses. Indeed, a significant difference was found in the used number of pulses between arms with additional prefrontal stimulation (3056.25 ± 871.78) and those with only temporal rTMS (1672.28 ± 723.44), U = 108.50, p < 0.01. As pointed out in Table 1, a *χ*^2^-test found a significant association between the use of an additional DLPFC stimulation and whether or not the effect of rTMS was significant. Since 80.70% of the study arms without an additional stimulation of the DLPFC report significant results, whereas only 56.25% of the study arms with an additional stimulation show significant effects, our findings suggest no benefit of an additional DLPFC stimulation, rather the opposite seems to pertain.

**Figure 3:**
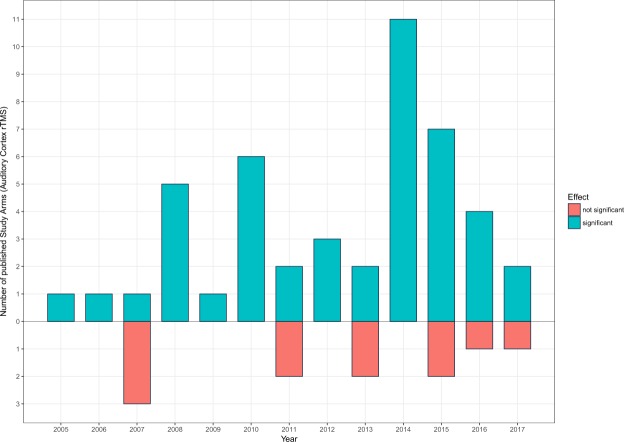
Year & Number of published Study Arms. For each year, the number of published study arms with an exclusively auditory cortex rTMS grouped for significant and not significant reported effects is illustrated.

In order to preclude a potential publication bias caused by a possible high quantity of significant or not significant results published in certain years (e.g., more significant studies in early years and more not significant studies in late years^36^), publication years of 57 study arms with solely auditory cortex rTMS were considered for statistical analysis to check if there is a mean difference between significant and not significant effects. No significant difference was found, perpending an exclusion of a publication bias, U = 241.50, p = 0.82. Figure 3 presents a summary of the quantity of published auditory cortex rTMS trial arms for each year subdivided by the reported effect.

All other parametric parameters were not significant. Mean motor threshold was not analyzed due to more than 30% of missing data (n = 65).

Analysis of categorical data showed no significant associations e.g. between the manufacturer of the TMS device (Magstim vs. MagVenture/Medtronic; number of other manufacturers were too low to include in analysis) or the default waveform of the system (biphasic cosine vs. biphasic sine). Coil orientation was not analyzed, because it was not reported in 34 study arms. Likewise, the type of coil was not included in statistical analysis, since 93.2% of examined arms used a figure-of-eight coil. Table 2 provides an overview over the missing information for all parameters separated for significant and not significant study arms.

**Table 2:** Missing data for rTMS parameters of 74 study arms.

rTMS Parameter	Significant Study Arms	Not Significant Study Arms
	N	N
Manufacturer TMS device	—	—
Pulse waveform	56	18
Default waveform	—	—
Coil type	—	1
Coil orientation	22	12
Stimulation position	1	—
Stimulation hemisphere	—	1
Stimulation frequency (%)	1	—
Stimulation intensity (%)	5	1
Mean motor threshold (%)	50	15
Motor threshold determination method	10	3
Number of sessions	1	—
Overall pulses per session over auditory and non—auditory cortical areas	1	—
Neuronavigation	—	—

## Discussion

Due to ongoing discussions about the effectiveness of rTMS in chronic tinnitus and recently initiated considerations if beside methodological also technical parameters of rTMS affect treatment efficacy, we conducted a systematic review of previous research concerning daily rTMS in tinnitus with the aim to present a statistical overview of general effectiveness of rTMS and to identify the influence of rTMS parameters on the consequences of verum rTMS in tinnitus.

With respect to the question if rTMS is generally effective as a treatment in tinnitus, we demonstrated that the proportion of significant pre-post comparisons is significantly higher for verum in contrast to sham arms. The chosen statistical strategy with observed effects dichotomized to “significant” and “not significant” is limited. Meta-analyses are rather suitable as a statistical approach to resolve this question. Furthermore, the quantity of eligible studies might be too low for valid analysis at this stage. Despite two recently published large trials with contradictory findings^24,25^, the debate on recommendation of auditory cortex rTMS for the treatment of chronic tinnitus is still not completed.

We explored two main associations of technical parameters on rTMS efficacy. First, we found that a lower stimulation intensity was associated with significance in the investigated study arms. This finding is in contrast to earlier work in depression and basic research concerning the motor cortex, which suggested a linear dose-response relationship, e.g. better treatment response^34,37^ or an increased influence on motor evoked potentials (MEPs)^33,38^ is associated with higher stimulation intensity. A clarification of the detected reversed impact appears to be difficult. One feasible explanation for this could be, that the stimulation intensity generates the intended consequences only up to a certain extent and then the effect either disappears or inverts. Similar changes are observed by the use of cTBS, which is also known to generate inhibitory effects such as 1 Hz rTMS. Applied at higher stimulation intensities, the inhibitory effects shift to excitatory^39,40^. These observations somewhat corroborate our findings, although with our collected data and statistical analysis we are not able to make a statement about possible excitatory effects of high stimulation intensities.

A further possible explanation could be related to skull thickness. Compared to the rest of the cranium, the average bone thickness over temporal parts is described as the thinnest^41^. Due to the location of the primary motor cortex under thicker bones, namely the interface of the frontal and parietal osseous, the motor threshold at this position might be an insufficient point of reference for the determination of the stimulation intensity for other stimulation positions. With respect to our results, a high intensity rTMS over thinner temporal bones might result in some kind of hyperstimulation which may induce contrary effects as argued above. Higher stimulation intensity potentially caused by visual determination of the motor threshold^42^ as well as a lower intensities caused by cTBS were excluded as shown by our analysis (cf. results section).

Secondly, the present systematic review indicates, that the lower number of pulses, including auditory and prefrontal cortical areas (per session and also for the whole trial), the more significant study arms were found. With regard to TBS, past research already investigated the effects of longer stimulation protocols with the insight that a prolongation of the stimulation per se does not lead to an improvement. A doubling of the stimulation length induced reversed after-effects e.g. inhibitory became excitatory^43^. For rTMS, a meta-analysis reported similar results, indicating a smaller number of pulses per session related to antidepressant mechanism of action^44^. The authors of this meta-analysis refer to other conducted meta-analyses with no such association – the key role of the quantity of pulses remains to be clarified. The same applies for the field of tinnitus. Former studies observed a substantial improvement in tinnitus-related outcome measurements with the usage of a higher number of pulses^45,46^. In contrast our investigation suggests the complete opposite.

It is very probable, that the effect is conceivably caused by an additional stimulation of the DLPFC, which features significant more pulses per session (cf. results section). Based on the rationale that via prefrontal rTMS anti-depressant effects take place^21^, an assumed interplay of tinnitus and depression^47,48^ and the involvement of prefrontal areas in auditory gating and tinnitus, combined frontal and temporal rTMS was proposed for more efficient suppression of tinnitus symptoms^49–51^. In contrast, our results are not in accordance with these findings. Addition of prefrontal rTMS eventuated in a significantly lower percentage of significant effects. In tinnitus, not only prefrontal areas, rather several cortical regions are involved, suggesting a widely spread network and also interindividual network profiles^52–55^. This postulation of several involved regions offers a putative approach to explain the unfavorable effect of additional prefrontal rTMS as only a “prefrontal” subtype would best benefit from this treatment and in other subtypes it might be contraindicative. Our finding of better effects of merely temporal stimulation provides support for the notion, that the final common pathway of tinnitus related pathophysiological alterations might still be the auditory cortex^56^.

One might argue that initial positive findings of solely auditory cortex stimulation motivated the field to concentrate on this stimulation protocol and might have induced a publication bias in the sense that initial clinical trials are often reported as promising with large effect sizes followed by years of increasing frustration showing a decrease of effect sizes and an increase of negative trials. Our analysis indicates that there is no change in the number of significant studies published per year (see Fig. 3) not revealing a potential publication bias with a time trend.

Due to the limited amount of data we were only able to analyze the effects of single rTMS parameters alone. However, we are aware of the possibility of specific interactions of several parameters, as demonstrated by pulse-quantity-dependent after-effects of excitatory intermittent theta burst stimulation (iTBS) on MEPS to be more distinct after lower stimulation intensities^57^. Likewise, it may be possible, that the inequality in the number of significant (n = 56) and not significant (n = 18) study arms bears an influence on our results.

Howsoever, the role of stimulation intensities and pulses per session for the effectiveness of rTMS needs to be systematically examined to clarify the outstanding issues of dose-dependent effects in tinnitus trials, especially as these findings are contradictory to the experiences of rTMS for the treatment of depression^58^. One of the initial intentions of this review was to investigate the effect of different waveforms on the effectiveness of rTMS. Unfortunately, not a single study reported the used pulse waveform for the stimulation. We have therefore decided to statistically analyze the default waveform settings of the device. We found no significant association between the type of biphasic waveform and the significance level of the effect. It was stated, that the coil orientation and the related induced direction of the currents are crucial in rTMS^59–61^. Biphasic pulses appear to be stronger, when passing the area of interest in anterior-posterior direction^28^. Due to many missing in the reported data, it was not possible to analyze the critical parameter current direction.

With the emerge of new technical innovations in the field of brain stimulation, exact stimulation positions via TMS integrated neuronavigation systems became a standard procedure. This review intended to determine the benefits of TMS neuronavigation systems in tinnitus. No such benefit is implied by our results. Either the local precision of stimulation does not play such a big role as presumed, or the targets were not optimally selected. Technical parameters like intensity or pulses, with neural mechanism not entirely understood, seem to be more important.

Several considerable methodological disadvantages were observed in the course of this review. A very important parameter in order to compare outcomes of different studies and even specify the effectiveness of a specific intervention is the definition of a primary outcome. We identified a lack of this information in N = 36 study arms of interest, leading to an adapted definition of observed significance for our review. In the course of examining appropriate research papers, unexpected differences (e.g., breaks during stimulation) within the methodology of 1 Hz rTMS were observed in some of the studies^62–67^. Such conditions make a comparison of trials even more difficult and introduce noise to the data. A major insight of the present work is the lack of reported essential rTMS parameters in the literature. Not only the full information about the used waveform was missing, but also relevant data on coil orientation or mean motor threshold features many missing values (cf. Table 2), which restricted our analysis. Guidelines for reporting e.g. the interventional methods used in clinical trials^68^ or a checklist for reporting parameters when deploying TMS on the motor system^69^ already exist. A paper from Wilson & St George^70^ and a recent review focusing on rTMS in the context of depression^71^ already emphasize the need of fully reported methodological information. The latter even provides a checklist for reporting rTMS parameters. Since both checklists enumerate essential parameters and details for rTMS, we strongly recommend their usage in future studies, to prospectively ensure a more precise and fundamental comparison of non-invasive brain stimulation studies using rTMS.

## Conclusion

The present systematic review demonstrates a higher efficacy of verum rTMS in contrast to sham rTMS. In verum arms, technical parameters such as stimulation intensity and number of pulses or restrictive stimulation of the auditory cortex were identified as relevant factors for clinical efficacy in a dose-dependent manner – less might be more. The impact of technical parameters in interaction with neurophysiological parameters (e.g., brain state before stimulation^72–74^) highlights the capability of rTMS in treating chronic tinnitus based on the premise to identify optimal stimulation protocols for single patients by means of personalized medical approaches^75^. In order to understand the consequences of considerable rTMS parameters in detail, standardized and sufficient reporting is highly required. As of yet, this is not the case – neither in tinnitus research nor in any other field utilizing rTMS^24,25,45,49–51,62–67,75–119^.

## Supplementary information

Supplemental Material 1

Supplemental Material 2
